# Kinematic measures of Arm-trunk movements during unilateral and bilateral reaching predict clinically important change in perceived arm use in daily activities after intensive stroke rehabilitation

**DOI:** 10.1186/s12984-015-0075-8

**Published:** 2015-09-21

**Authors:** Hao-ling Chen, Keh-chung Lin, Rong-jiuan Liing, Ching-yi Wu, Chia-ling Chen

**Affiliations:** School of Occupational Therapy, College of Medicine, National Taiwan University, Taipei, Taiwan; Division of Occupational Therapy, Department of Physical Medicine and Rehabilitation, National Taiwan University Hospital, Taipei, Taiwan; Department of Occupational Therapy and Graduate Institute of Behavioral Sciences, College of Medicine, Chang Gung University, Taoyuan, Taiwan; Healthy Ageing Research Center, Chang Gung University, Taoyuan, Taiwan; Department of Physical Medicine and Rehabilitation, Chang Gung Memorial Hospital, Taoyuan, Taiwan

**Keywords:** Kinematics, Reaching, Stroke, Clinically important change, Daily function

## Abstract

**Background:**

Kinematic analysis has been used to objectively evaluate movement patterns, quality, and strategies during reaching tasks. However, no study has investigated whether kinematic variables during unilateral and bilateral reaching tasks predict a patient’s perceived arm use during activities of daily living (ADL) after an intensive intervention. Therefore, this study investigated whether kinematic measures during unilateral and bilateral reaching tasks before an intervention can predict clinically meaningful improvement in perceived arm use during ADL after intensive poststroke rehabilitation.

**Methods:**

The study was a secondary analysis of 120 subjects with chronic stroke who received 90–120 min of intensive intervention every weekday for 3–4 weeks. Reaching kinematics during unilateral and bilateral tasks and the Motor Activity Log (MAL) were evaluated before and after the intervention.

**Results:**

Kinematic variables explained 22 and 11 % of the variance in actual amount of use (AOU) and quality of movement (QOM), respectively, of MAL improvement during unilateral reaching tasks. Kinematic variables also explained 21 and 31 % of the variance in MAL-AOU and MAL-QOM, respectively, during bilateral reaching tasks. Selected kinematic variables, including endpoint variables, trunk involvement, and joint recruitment and interjoint coordination, were significant predictors for improvement in perceived arm use during ADL (*P* < 0.05).

**Conclusions:**

Arm–trunk kinematics may be used to predict clinically meaningful improvement in perceived arm use during ADL after intensive rehabilitation. Involvement of interjoint coordination and trunk control variables as predictors in bilateral reaching models indicates that a high level of motor control (i.e., multijoint coordination) and trunk stability may be important in obtaining treatment gains in arm use, especially for bilateral daily activities, in intensive rehabilitation after stroke.

## Background

Upper extremity (UE) hemiparesis is a major residual deficit in patients with stroke [1, 2]. The inability to incorporate the affected arm into daily activities may limit a patient’s independence in the community [3, 4]; therefore, UE motor training has become an important goal of stroke rehabilitation. Numerous clinical measurements have been used to evaluate the improvement of motor performance after rehabilitation [5–7]. The use of a Likert scale by multiple raters might result in measurement variability that might mask the demonstration of real treatment effects [8]. A reliable, repeatable assessment of continuous measures of impairment and treatment change has been called for [9]. Kinematic analysis provides a valuable, objective evaluation of motor performance during functional tasks and offers information on movement patterns, quality, and strategies [10–12]. This study investigated whether reaching kinematics before an intervention can predict functional improvements after intensive rehabilitation.

Reaching, a fundamental element of many activities of daily living (ADL), has often been chosen as the represented task for kinematic measures [13]. Kinematic variables, including movement trajectories (endpoint control), joint recruitment and interjoint coordination, and trunk involvement, are frequently used to characterize the deficit, recovery, and treatment effects of control strategies during reaching after stroke [12, 14–17]. Patients with stroke demonstrate deficits in endpoint control and disrupted joint recruitment and interjoint coordination. Impaired endpoint control is characterized by smaller movement amplitude, prolonged movement times, and more segmented movement trajectories [18–20]. Patients after stroke may also recruit new degrees of freedom such as trunk involvement to accomplish goal-directed reaching tasks [11, 21, 22]. After-stroke interventions (e.g., constraint-induced therapy, robot-assisted arm training), recovered endpoint control (e.g., better temporal efficiency and smoothness) and joint recruitment patterns (e.g., more shoulder flexion and elbow extension, less trunk compensation) have been found [12, 14–17].

To aid using kinematic data during reaching tasks to function as outcome measures, a better understanding of the nature of the kinematics in relation to movement output, as measured by clinical scales, is necessary [23–25]. Previous studies showed significant relationships between kinematic variables and sensorimotor impairments and activity capacity limitation of the UE [9, 13, 26, 27]. For example, the endpoint variable of peak velocity, representing force control strategy, was correlated with UE strength and active range of motion [13]. Elbow extension recruitment [27] and trunk involvement [26] during reaching tasks can reflect the sensorimotor impairments of the UE measured by the Fugl-Meyer Assessment (FMA) and the Wolf Motor Function Test (WMFT) in patients with stroke.

Murphy et al. [9] first studied the relation of kinematic variables to self-perceived activity performance in daily life measured by ABILHAND and found that the movement unit variable (smoothness of movement) explained 6 % of the variance in ABILHAND. This small variance was possibly due to the ABILHAND assessment scale. The ABILHAND questionnaire evaluates patients’ perceived difficulties in performing unimanual or bimanual tasks, which might include many contextual aspects rather than movement skills per se [9]. For example, one question asks indicates whether the patient can fasten a zipper on a jacket without technical or human help. Other clinical scales of self-perceived activity performance, such as the Motor Activity Log (MAL), which measures patients’ perceived use of the more-affected arm during ADL and which emphasize motor aspects during ADL (i.e., the amount and quality of movement performed), may be more related to kinematic variables than ABILHAND.

Kinematic measures bear a significant relationship to clinical measures of sensorimotor impairment and activity capacity limitation and can detect treatment effects on motor control in patients with stroke [12, 14–17]. In the course of recovery after stroke, the FMA score improved, with increases in movement smoothness (data from 13 patients receiving motor rehabilitation) [28], and changes in endpoint trajectories and trunk involvement were significantly associated with changes in activity capacity evaluated by the Action Research Arm Test (ARAT) (data from 51 patients receiving standard rehabilitation) [29]. However, Massie et al. [27] pointed out the need to further elucidate whether kinematic performance at baseline allows for predictions of sensorimotor impairments, activity capacity, and performance limitation over time. To date, no study has examined whether kinematic variables during reaching tasks predict perceived arm use during ADL after a UE intervention.

The FMA and ARAT evaluate the sensorimotor impairment or activity capacity limitation under a “standardized” environment [30], which describes a patient’s highest probable level of functioning rather than the patient’s actual activity performance in daily life. Evaluation of self-perceived activity performance can provide more information on how the intervention’s effects could be generalized to the home or community environment [3]. Moreover, because patient-reported outcomes were emphasized as the primary choice for evidence-based rehabilitation [31], MAL, a questionnaire measuring self-perception has been found suitable for ADL assessment in patients with stroke after intensive rehabilitation.

Kinematics may be an important or added index for effective evaluation of arm use during ADL after stroke rehabilitation, but the predictive accuracy of kinematic measures on the perceived arm use during ADL is not well understood. Therefore, the present study investigated whether the kinematic measures during reaching tasks before an intervention can predict clinically meaningful improvement on perceived arm use during ADL, measured by MAL, in patients with stroke after intensive stroke rehabilitation. Instead of considering the amount of improvement, the present study emphasized improvement that was clinically important and perceived as beneficial by the patients. This study also sought kinematic variables important for predicting the proportion of patients with stroke that could make a clinically meaningful improvement in perceived arm use during ADL. Because ADL can be accomplished by unilateral or bilateral UE, the study analyzed arm–trunk kinematic predictors, namely, endpoint control, joint recruitment and interjoint coordination, and trunk involvement, during both unilateral and bilateral reaching tasks.

## Methods

### Participants

This study was a secondary analysis of data obtained from previous and ongoing randomized controlled trials of intensive training, including constraint-induced therapy (CIT), bilateral arm training (BAT), robot-assisted arm training (RT), mirror therapy (MT), and conventional training (CT) [10, 16, 32, 33]. Data from 120 stroke patients were analyzed; namely, 14 for CIT, 10 for BAT, 41 for RT, 37 for MT, and 18 for CT. The inclusion criteria of this study were (1) at least 6 months after onset from a unilateral stroke, (2) Brunnstrom stage of the UE ≥ III, and (3) being able to follow instructions during the evaluation and intervention (mini mental state examination ≥ 24). The exclusion criteria were (1) excessive spasticity at any UE joint (Modified Ashworth Scale score > 2), (2) receiving experimental rehabilitation or drug treatment within the past 3 months, and (3) an additional neurologic condition or health problem that might affect the effects of intervention.

This study was approved by the Institutional Review Board at each participating site. All participants signed informed consent forms in which primary and secondary analysis of the obtained data are included. The demographic characteristics of the participants are presented in Table 1.

**Table 1:** Demographics and clinical characteristics of the participants (*N* = 120)

Characteristics	Value
Age, mean ± SD, years	53.90 ± 10.38
Time after stroke, mean ± SD, months	20.02 ± 14.58
Sex, No. (%)	
Male	85 (71)
Female	35 (29)
Side of stroke, No. (%)	
Right	57 (47.5)
Left	63 (52.5)
Stroke type, No. (%)	
Ischemic	65 (54.17)
Hemorrhagic	55 (48.83)
FMA score (UE total score: 66), mean ± SD	42.95 ± 9.20

*FMA* Fugl-Meyer Assessment, *SD*, standard deviation

### Procedures

Participants completed one of the interventions, namely, CIT, BAT, RT, MT, or CT, for 90–120 min every weekday for 3–4 weeks. The outcome measure (MAL) was administered twice to each patient by the same rater, once before the intervention and again after the intervention. Six trained raters, blinded to the participant’s group, performed the clinical evaluations (i.e., MAL).

### Outcome measures

The MAL, a semistructured interview questionnaire [34, 35], was used to assess the patient’s perception of actual amount of use (AOU) and quality of movement (QOM) of the patient’s affected arm during ADL outside the treatment setting. The MAL consists of 30 common activities involving the UE, such as opening a drawer, getting up from a chair with armrests, putting on socks, and picking up a cup by the handle [36]. The scores range from 0 (never or incapability to use the affected arm) to 5 (the ability to use the affected arm was as good as before the stroke). The MAL was originally designed for patients with hemiparetic stroke and has frequently been used to evaluate the effectiveness of interventions such as CIT and BAT [10, 37, 38]. The MAL has shown good reliability (*r* = 0.82) and concurrent validity with the Wolf Motor Function Test (Spearman *ρ* = 0.64–0.99) [39, 40].

Minimal clinically important changes, defined as the smallest change in an outcome measure perceived as beneficial to patients, can provide more information for clarification of clinically meaningful changes and is often used as a threshold of meaningful improvement after treatment [41, 42]. On the basis of clinical experience, estimates reported in the literature using MAL, [35, 43, 44] and other outcome measures, such as ARAT, reflecting activity capacity limitation [45], and Barthel Index, reflecting activity performance limitation [46] for patients with stroke, we set the minimal clinically important changes on the MAL-AOU and MAL-QOM at 10 % of the scale scores (i.e., 0.5) in the present study. A mean improvement in the MAL-AOU and MAL-QOM score greater than 0.5 may indicate that the patient perceives the improvement of the AOU or QOM of the affected arm during ADL.

### Potential predictors

Twelve potential predictors (arm–trunk kinematics) were obtained before intervention from the functional tasks requiring reaching. The reaching tasks included a unilateral task of reaching to press a desk bell using the affected arm and a bilateral task in which both arms simultaneously reached to press desk bells. During the unilateral task, the desk bell was placed along the participants’ midsagittal plane at arm’s length, defined as the distance between the medial border of the axilla and the midpoint of the styloid processes of ulna and radius. During the bilateral task, each desk bell was placed in front of each arm at arm’s length. Each participant had one practice trial to become familiar with the task. The participant was then instructed to reach and press the desk bell as quickly as possible, after hearing a beep signal, for three successful trials. The trunk was not constrained during the reaching tasks, and trunk motion was allowed.

Nineteen markers were placed on the UE, including the spinal processes of the seventh cervical vertebra (C7) and fourth thoracic vertebra (T4), midsternum, bilateral clavicular heads, acromions, the middle of the humeri, lateral epicondyles, styloid processes of ulna and radius, thumbnails, and the nails of the index fingers. Three-dimensional marker trajectories were measured with a seven-camera motion analysis system (VICON MX, Oxford Metrics Inc, Oxford, UK) at a sampling rate of 120 Hz and were filtered using a second-order low-pass Butterworth filter with a cutoff frequency of 5 Hz. A customized LabVIEW program (National Instruments Inc, Austin, TX) was used to calculate arm–trunk kinematics. The kinematic model applied in this study had good reliability (intraclass correlation coefficient = 0.74–0.95) [17].

Reaching to press a desk bell requires precise endpoint control through highly coordinated joint movements of all UE joints. Trunk compensatory movement was commonly found in stroke patients during reaching. Therefore, three kinds of arm–trunk kinematic variables were chosen to describe the arm–trunk motor control strategies during reaching: endpoint variables; UE joint recruitment and interjoint coordination variables; and trunk movement variables. Endpoint variables, calculated according to the marker on the nail of the index finger (endpoint), included reaction time (EndRT), movement time (EndMT), peak velocity (EndPV), the percentage of EndMT when the EndPV occurred (EndPPV), and movement unit (EndMU). EndRT is the interval between onset of signal and movement, whereas EndMT is the interval between movement onset and offset. The movement onset was defined for each trial as the time at which the tangential velocity rose above baseline by 5 % of the peak tangential velocity of the index marker, and movement offset was defined as the time at which the tangential velocity fell and remained below 5 % of the peak tangential velocity. These temporally related variables represent the efficiency of motor preplanning and execution. EndPV and EndPPV represent the force control strategy during reaching. One EndMU consists of one acceleration phase and one deceleration phase. Fewer EndMUs indicate smoother movement.

UE joint recruitment and interjoint coordination variables included maximal angles of shoulder flexion (SFlex), shoulder abduction (SAbd), and elbow extension (EExt), as well as the values and time when maximal cross correlation between shoulder flexion and elbow extension occurred (S-ECC, TS-ECC). S-ECC represents the maximum similarity of the time-angle waveforms of shoulder flexion and elbow extension as a function of a time lag applied to the initiation of elbow movement. Higher S-ECC values indicate better interjoint coordination between the shoulder and elbow.

Trunk involvement variables included maximal trunk flexion (TFlex) and maximal trunk lateral shift displacement to the sound side (TSS). TFlex was calculated as the angle between the vector joining the C7–T4 markers and the vector parallel to the direction of gravity. Negative TSS values indicate lateral shift of the trunk to the affected side.

### Data analysis

The Wilcoxon signed rank test was used to compare the MAL score between pretreatment and posttreatment. Multiple logistic regression analyses were conducted separately for unilateral and bilateral reaching to identify predictors that could contribute to predicting clinically important changes on the MAL-AOU and MAL-QOM models. Participants whose improvement on the MAL-AOU or MAL-QOM exceeded minimal clinically important changes (≥0.5) were classified as the group with positive change (coded 1), whereas participants who did not reach this criterion were in the group with no positive change (coded 0). Multicollinearity among the predictors was tested by the criterion of a variance inflation factor (VIF) higher than 10. Predictors for which the VIF exceeded 10 were excluded from the models.

To ensure the quality of the results, the Hosmer-Lemeshow goodness-of-fit test was used for the logistic regression models. A Hosmer-Lemeshow test *P* value > 0.05 suggests that the model fits the data closely. The Nagelkerke *R*^2^ was used for variance of the explained measure. A Nagelkerke *R*^2^ between 0.10 and 0.20 is considered satisfactory, and values between 0.20 and 0.40 are very satisfactory [47, 48]. Odds ratios of the significant predictors were generated from the analyses. A significance level was set at 0.05. Statistical analyses were performed using SPSS 19.0 software (IBM Corp, Armonk, NY).

## Results

The results of the Wilcoxon signed rank test showed that the scores of our outcome measures were significantly increased at posttreatment (MAL-AOU: 1.26 ± 0.94, MAL-QOM: 1.27 ± 0.90) compared with pretreatment (MAL-AOU: 0.78 ± 0.82, MAL-QOM: 0.84 ± 0.83; *P* < 0.001) in the patients with stroke. Moreover, 45.83 % (55/120) and 38.33 % (46/120) of the participants reached a clinically important change (coded 1) in MAL-AOU and MAL-QOM, respectively. For the arm–trunk kinematic predictors during unilateral reaching tasks, the results of logistic regression analysis showed that EndRT, SFlex, SAbd, and EExt were significant predictors of clinically important changes on MAL-AOU (Nagelkerke *R*^2^ = 0.22, Table 2). EndPV was the only significant predictor of clinically important changes on MAL-QOM (Nagelkerke *R*^2^ = 0.11, Table 2).

**Table 2:** Logistic regression analyses for clinically important changes on the Motor Activity Log (MAL)

Predictors	MAL-AOU				MAL-QOM			
	β	Wald [56]	P	OR (95 % CI)	β	Wald [56]	P	OR (95 % CI)
Unilateral Task								
Constant	5.84				1.64			
EndRT (ms)	0.004	7.64	<0.01	1.004 (1.001–1.01)				
EndPV (m/s)					−3.03	8.18	<0.01	0.05 (0.01–0.39)
SAbd (degree)	−0.06	5.60	0.018	0.94 (0.89–0.99)				
SFlex (degree)	0.06	5.29	0.021	1.06 (1.01–1.11)				
EExt (degree)	−0.08	11.11	<0.01	0.92 (0.88–0.97)				
Nagelkerke R^2^	0.22				0.11			
−2 Log likelihood	144.11				150.09			
Bilateral Task								
Constant	4.40				2.48			
EndRT (ms)	0.003	4.62	0.032	1.003 (1.00–1.01)	0.003	3.95	0.047	1.003 (1.0–1.01)
EndMT (s)	−0.53	4.06	0.044	0.59 (0.35–0.99)	−0.64	4.40	0.036	0.53 (0.29–0.96)
EndPV (m/s)					−4.45	10.41	<0.01	0.01 (0.001–0.18)
S-ECC	−5.01	10.62	<0.01	0.01 (0–0.14)				
TFlex	−0.1	6.44	0.011	0.90 (0.83–0.98)	−0.07	4.26	0.039	0.94 (0.88–0.10)
TSS (mm)					0.05	7.82	<0.01	1.05 (1.01–1.08)
Nagelkerke R^2^	0.21				0.31			
−2 Log likelihood	144.93				128.90			

Only significant predictors for affected limb before intervention are reported

*MAL-AOU* amount of actual amount of use in MAL, *MAL-QOM* quality of movement in MAL, *EndRT* endpoint reaction time, *EndMT* endpoint movement time, *EndPV* endpoint peak velocity, *SFlex* maximal shoulder flexion, *SAbd* maximal shoulder abduction, *EExt* maximal elbow extension, *S-ECC* maximal cross correlation between shoulder flexion and elbow extension, *TFlex* maximal trunk flexion, *TSS* maximal trunk lateral shift displacement to the sound side, *β* estimated coefficient, *Wald* Wald statistics, *CI* confidence interval, *OR* odds ratio

The logistic regression equations for the unilateral task are as follows:

$$ \mathrm{LogitP}\ {\left(\mathrm{change}\ \mathrm{score}\ \ge\ 0.5\ \mathrm{o}\mathrm{n}\ \mathrm{t}\mathrm{h}\mathrm{e}\ \mathrm{M}\mathrm{A}\mathrm{L}\hbox{-} \mathrm{A}\mathrm{O}\mathrm{U}\right)}_{\mathrm{unilateral}} = 5.84 + 0.004 \times \mathrm{E}\mathrm{n}\mathrm{d}\mathrm{R}\mathrm{T} + 0.06 \times \mathrm{S}\mathrm{F}\mathrm{l}\mathrm{e}\mathrm{x}\ \hbox{-}\ 0.08 \times \mathrm{E}\mathrm{E}\mathrm{x}\mathrm{t}\ \hbox{-}\ 0.06 \times \mathrm{S}\mathrm{A}\mathrm{bd} $$

$$ \mathrm{LogitP}\ {\left(\mathrm{c}\mathrm{h}\mathrm{a}\mathrm{n}\mathrm{g}\mathrm{e}\ \mathrm{scor}\mathrm{e}\ \ge\ 0.5\ \mathrm{o}\mathrm{n}\ \mathrm{t}\mathrm{h}\mathrm{e}\ \mathrm{M}\mathrm{A}\mathrm{L}\hbox{-} \mathrm{Q}\mathrm{O}\mathrm{M}\right)}_{\mathrm{unilateral}} = 1.64\ \hbox{-}\ 3.03 \times \mathrm{E}\mathrm{n}\mathrm{d}\mathrm{P}\mathrm{V} $$

As determined from these equations and odds ratio estimates (Table 2), a 1-unit increase in the baseline EndRT (ms) or SFlex (degrees) during unilateral reaching led to a 1.004-times or 1.06-times higher probability, respectively, of achieving a clinically important change in MAL-AOU. If the increase in the baseline EndRT or SFlex was 100 ms or 10°, the odds of achieving a clinically important change in MAL-AOU increased 1.49 times or 1.8 times*,* respectively. However, for a 1-unit increase in the baseline SAbd (degrees) or EExt (degrees) during unilateral reaching, the odds of achieving a clinically important change in MAL-AOU decreased from 1 to 0.94 or from 1 to 0.92, respectively. In other words, with a 1-unit decrease in the baseline SAbd or EExt during unilateral reaching, the odds of achieving clinically important changes in MAL-AOU increased 1.06 times or 1.08 times*,* respectively. If the decrease in the baseline SAbd or EExt was 10°, the odds of achieving clinically important changes in MAL-AOU increased 1.82 times or 2.23 times, respectively. However, for a 1-unit increase in the baseline EndPV (m/s) during unilateral reaching, the odds of achieving a clinically important change in MAL-QOM decreased from 1 to 0.05. This means that a 1-unit decrease in the baseline EndPV during unilateral reaching led to 20.7-times higher probability of achieving clinically important changes in MAL-QOM.

For the arm–trunk kinematic predictors during bilateral reaching tasks, EndRT, EndMT, S-ECC, and TFlex were selected into the MAL-AOU model (Nagelkerke *R*^2^ = 0.21, Table 2). EndRT, EndMT, EndPV, TFlex, and TSS were selected into the MAL-QOM model (Nagelkerke *R*^2^ = 0.31, Table 2).

The logistic regression equations for the bilateral task are as follows:

$$ \mathrm{LogitP}\ {\left(\mathrm{c}\mathrm{h}\mathrm{a}\mathrm{n}\mathrm{g}\mathrm{e}\ \mathrm{scor}\mathrm{e}\ \ge\ 0.5\ \mathrm{o}\mathrm{n}\ \mathrm{t}\mathrm{h}\mathrm{e}\ \mathrm{M}\mathrm{A}\mathrm{L}\hbox{-} \mathrm{A}\mathrm{O}\mathrm{U}\right)}_{\mathrm{bilateral}} = 4.40 + 0.003 \times \mathrm{E}\mathrm{n}\mathrm{d}\mathrm{R}\mathrm{T}\ \hbox{-}\ 0.53 \times \mathrm{E}\mathrm{n}\mathrm{d}\mathrm{M}\mathrm{T}\ \hbox{-}\ 5.01 \times \mathrm{S}\hbox{-} \mathrm{E}\mathrm{C}\mathrm{C}\ \hbox{-}\ 0.1 \times \mathrm{T}\mathrm{F}\mathrm{l}\mathrm{e}\mathrm{x} $$

$$ \mathrm{LogitP}\ {\left(\mathrm{c}\mathrm{h}\mathrm{a}\mathrm{n}\mathrm{g}\mathrm{e}\ \mathrm{scor}\mathrm{e}\ \ge\ 0.5\ \mathrm{o}\mathrm{n}\ \mathrm{t}\mathrm{h}\mathrm{e}\ \mathrm{M}\mathrm{A}\mathrm{L}\hbox{-} \mathrm{Q}\mathrm{O}\mathrm{M}\right)}_{\mathrm{bilateral}} = 2.48 + 0.003 \times \mathrm{E}\mathrm{n}\mathrm{d}\mathrm{R}\mathrm{T} + 0.05 \times \mathrm{T}\mathrm{S}\mathrm{S}\ \hbox{-}\ 0.64 \times \mathrm{E}\mathrm{n}\mathrm{d}\mathrm{M}\mathrm{T}\ \hbox{-}\ 4.45 \times \mathrm{E}\mathrm{n}\mathrm{d}\mathrm{P}\mathrm{V}\ \hbox{-}\ 0.07 \times \mathrm{T}\mathrm{F}\mathrm{l}\mathrm{e}\mathrm{x} $$

As determined from these equations and odds ratio estimates (Table 2), a 1-unit increase in the baseline EndRT during bilateral reaching led to a 1.003-times higher probability of achieving a clinically important change in MAL-AOU. If the increase in the baseline EndRT was 100 ms, the odds of achieving clinically important changes in MAL-AOU increased 1.35 times. However, for a 1-unit increase in the baseline EndMT (s), S-ECC (points), or TFlex (degrees) during bilateral reaching, the odds of achieving clinically important changes in MAL-AOU decreased from 1 to 0.59, 1 to 0.01, or 1 to 0.9, respectively. In other words, 1-unit decrease in the baseline EndMT, S-ECC, or TFlex during bilateral reaching led to a 1.70-times, 149.90-times, or 1.11-times higher probability, respectively, of achieving clinically important changes in MAL-QOM. Moreover, a 1-unit increase in the baseline EndRT or TSS (mm) during bilateral reaching led to a 1.003-times or 1.05-times higher probability, respectively, of achieving a clinically important change in MAL-QOM. For a 1-unit increase in the baseline EndMT, EndPV, and TFlex during bilateral reaching, the odds of achieving a clinically important change in MAL-QOM decreased from 1 to 0.53, 1 to 0.01, or 1 to 0.94, respectively. In other words, a 1-unit decrease in the baseline EndMT, EndPV, and TFlex during bilateral reaching led to a 1.90-times, 85.63-times, or 1.07-times higher probability, respectively, of achieving clinically important changes in MAL-QOM. If the increase in the baseline TFlex was 10°, the odds of achieving clinically important changes in MAL-QOM increased 2.01 times.

## Discussion

This study is the first to use the possible kinematic variables during reaching tasks to predict clinically meaningful improvement in perceived arm use during ADL, measured by MAL, in patients with stroke. Previous studies associated kinematic assessment of reaching movements with clinical scores [9, 13, 26, 27]. This study extends these previous findings to using kinematic variables as predictors for clinically important changes in functional outcomes after an intervention.

The results of this study suggest that kinematic measures during unilateral and bilateral reaching tasks have sufficient predictability for meaningful improvement of MAL, representing perceived arm use during ADL. Predictors of intervention outcome on MAL have been investigated extensively [5, 6, 49, 50]. However, only demographic and clinical scale data were used as potential predictors. Sufficient and comparable predictability of the kinematic measures found in the present study suggest that, apart from demographic and clinical scale data, selected kinematic variables are also important for the prognosis of perceived arm use during ADL after an intervention in patients with stroke. The results also suggest that selected kinematic variables might be considered simultaneously as predictors of MAL in future studies. Furthermore, a combination of different aspects of kinematic measures, such as endpoint control, UE recruitment, interjoint coordination, and trunk involvement, might be preferable to predict self-perceived functional outcomes after an intervention.

The *R*^2^ results show that predictive models of the bilateral reaching task explain more variance in clinically meaningful improvement of MAL, especially in MAL-QOM. The differential predictability of the models between unilateral and bilateral reaching may be attributed to the kinematic differences between unilateral and bilateral reaching. Because many natural ADL require bilateral movements [51], motor performance of the affected arm during a bilateral reaching task may provide more information reflecting the actual use of the affected arm during ADL. In addition, the role of the affected arm in patients with hemiplegic stroke may be to assist the sound arm rather than to independently implement activities, which is inconsistent with how the affected arm was used during the unilateral task. Although Murphy et al. [9] found that one of the endpoint control variables, movement unit, explained only 6 % of the variance in ABILHAND, the present study demonstrated that arm–trunk kinematics explain satisfactory variances of MAL. The different results may be due to the use of a cross-sectional design vs. a pretest-posttest study design and the clinical measures used for evaluating self-perceived activity performance (ABILHAND vs. MAL). Moreover, movement units were not a significant predictor in the present study. One possible reason is that the contribution of UE joint recruitment and interjoint coordination variables, which were not included in the Murphy et al. study but were included in the present study, was greater than that of movement unit, helping improve the predictability of self-perceived activity performance.

For both the unilateral and bilateral reaching models, time-related endpoint variables were predictors for MAL-AOU and MAL-QOM. Participants who demonstrate poor motor preplanning efficiency (i.e., longer EndRT) but good execution efficiency (i.e., shorter EndMT) before the intervention may have a higher probability of achieving clinically meaningful improvement in perceived arm use during ADL. On the one hand, patients with inefficient motor preplanning may have more room for improvement after intensive training. On the other hand, patients with the capacity to efficiently perform a task may have greater opportunities for task practice during a training program to improve motor skills and problem-solving abilities; accordingly, they may have a better prognosis in perceived arm use during ADL. This finding is particularly important for improving the utility of kinematic analysis in clinics, because time-related endpoint control variables (i.e., EndRT and EndMT) can be obtained easily in the clinic without a sophisticated motion analysis system.

For both the unilateral and bilateral reaching models, EndPV was a predictor only for MAL-QOM, considering the speed of the affected arm during ADL. Inclusion of EndPV, a speed-related kinematic variable, as a predictor for MAL-QOM may come from the speed-emphasized instruction during our reaching tasks. This finding indicated that the speed-emphasized instruction might cause kinematic variables, such as EndPV, to become a salient predictor. EndPV is associated with force generation, and appropriate force generation is related to smooth motor performance with less feedback correction [20]. Patients with poorer force control (i.e., lower EndPV) may have the potential to improve motor performance through motor training and develop self-perceived better quality of movement performed during ADL. For the unilateral reaching model, EndPV was the only predictor for MAL-QOM. Although the explained variance of this model is satisfactory, the variance explained (11 %) might be considered relatively low and the results from this model should be used with caution.

The UE recruitment and interjoint coordination variables were included in both the unilateral and bilateral reaching models for MAL-AOU. However, different predictors were included. UE recruitment, namely SAbd, SFlex, and EExt, were significant predictors for the unilateral reaching model, whereas the interjoint coordination variable (S-ECC), simultaneously considering shoulder and elbow joint recruitment, was a significant predictor for the bilateral reaching model. The differences in the predictors between unilateral and bilateral reaching models may lie in the joint control. It is possible that unilateral reaching requires elementary joint control to overcome pathological synergies (i.e., shoulder abduction or flexion and elbow flexion) [52]. However, bilateral reaching emphasizes bimanual coordination, possibly resulting in a higher-level variable of motor control [53] (i.e., shoulder–elbow coordination) as an important predictor. Efficacy studies [11, 54] suggest that intensive training might improve interjoint coordination. Patients with poor coupling in the affected arm’s shoulder flexion and elbow extension during bilateral reaching may benefit greatly from an intervention, with a corresponding good prognosis of perceived arm use during ADL.

Trunk involvement variables, TFlex and TSS, were predictors only for bilateral reaching models. The use of less trunk compensation (i.e., smaller TFlex) might lead to a greater use of the affected arm and quality of movement during functional activities. Symmetric posture (i.e., TSS close to zero) might also lead to better quality of movement. Using trunk compensatory strategies to achieve an immediate task goal may be detrimental to long-term functional recovery [55]. Patients relied on a trunk compensatory strategy to perform required tasks before receiving intensive intervention, which may hinder the attainment of treatment gains in perceived arm use during ADL, and decrease the opportunity and capacity to use the affected arm to perform tasks. Although a previous study [56] found that trunk control measured by a clinical scale is a predictor for ADL performance, this study, in terms of kinematic analysis, specifically pointed out the element of trunk control that included trunk flexion and lateral shift during bilateral reaching as a predictor of perceived arm use during ADL.

Nine kinematic predictors have been chosen to predict improvement of MAL. On the basis of the predictive models, a shorter EndMT and good initial motor performance without compensation (i.e., greater SFlex, greater TSS, smaller SAbd, and smaller TFlex) may result in a better prognosis for perceived arm use during ADL after intensive training. Initial movement without using a compensatory strategy may result in better treatment gains and increase the capacity of remediation of impairment. However, some kinematic predictors may have an opposite relationship with MAL improvement. A longer EndRT as well as lower EndPV, EExt, and S-ECC (i.e., poor initial levels) may lead to a better prognosis for perceived arm use during ADL. Poor initial levels but better prognosis associated with these kinematic variables may suggest that these kinematic variables have more room for improvement and may obtain beneficial effects from our intensively task-oriented motor training, which emphasized endpoint and joint control training. It seems that different kinematic predictors may play different roles in improvement of perceived arm use during ADL after intensive stroke rehabilitation. Moreover, the present study attempted to explore meaningful kinematic variables predicting treatment effects. Future research may investigate the minimal clinically important changes in kinematic variables to triage patients into clinical improvement or non-improvement groups, which may be helpful for determining the treatment programs with remedial or compensatory approaches.

### Limitations

This study has four limitations. First, only chronic patients with mild to moderate stroke were recruited; thus, predictors for perceived arm use during ADL found in the present study may not be generalized to stroke patients with different severity. Second, unilateral and bilateral reaching tasks with normal arm’s length were used. Different task conditions, such as reaching beyond arm’s length and a drinking task, might lead to different predictors for perceived arm use during ADL. Third, only kinematic predictors were considered as potential predictors for perceived arm use during ADL. Future research may consider incorporation of kinematic variables with treatment types, clinical measures, and participant characteristics, such as the initial level of MAL, to enhance the predictability of perceived arm use during ADL. Fourth, this study was designed as a secondary analysis. Future research might follow up with a new, prospective sample to validate the results of the present and previous studies.

## Conclusions

Arm–trunk kinematics may be used to predict clinically meaningful improvement of perceived arm use during ADL after an intervention. Three aspects of kinematic variables, including endpoint control variables, UE recruitment and interjoint coordination, as well as trunk involvement, are key elements in predicting clinically important functional outcomes. Involvement of interjoint coordination and trunk control variables as predictors in bilateral reaching models indicates that high levels of motor control (i.e., multijoint coordination) and trunk stability may be important in obtaining activity performance gains, especially for bilateral activities, in intensive rehabilitation after stroke.

## Abbreviations

MAL: Motor Activity Log
UE: Upper extremity
ADL: activities of daily living
FMA: Fugl-Meyer Assessment
ARAT: Action Research Arm Test
CIT: Constraint-Induced Therapy
BAT: Bilateral Arm Training
RT: Robot-assisted arm Training
MT: Mirror Therapy
CT: Conventional Training
AOU: Actual amount of use
QOM: Quality of movement
C7: Seventh cervical vertebra
T4: Fourth thoracic vertebra
EndRT: Endpoint reaction time
EndMT: Endpoint movement time
EndPV: Endpoint peak velocity
EndPPV: Percentage of EndMT of endpoint when the EndPV occurred
EndMU: Endpoint movement unit
SFlex: Maximal shoulder flexion
SAbd: Maximal shoulder abduction
EExt: Maximal elbow extension
S-ECC: Maximal cross correlation between shoulder flexion and elbow extension
TS-ECC: Time when maximal cross correlation between shoulder flexion and elbow extension occurred
TFlex: Maximal trunk flexion
TSS: Maximal trunk lateral shift displacement to the sound side
VIF: Variance inflation factor
